# Controlling room temperature ferromagnetism and band gap in ZnO nanostructured thin films by varying angle of implantation

**DOI:** 10.1039/c7ra10615g

**Published:** 2018-02-06

**Authors:** Rajesh V. Hariwal, Hitendra K. Malik, Ambika Negi, Asokan Kandasami

**Affiliations:** Inter-University Accelerator Centre Aruna Asaf Ali Marg New Delhi – 110067 India hariwal@gmail.com; Department of Physics, Indian Institute of Technology Delhi New Delhi-110016 India; Department of Physics, Acharya Narendra Dev College, University of Delhi New Delhi-110019 India

## Abstract

The defects in the host lattice play a major role in tuning the surface roughness, optical band gap and the room temperature ferromagnetism (RTFM) of ZnO thin films. Herein, we report a novel approach to tailor the band gap and RTFM of a ZnO nanostructure by varying the angle of implantation of 60 keV N ions keeping the ion fluence of 1 × 10^16^ ions per cm^2^ and the beam size of 3 mm constant. The implantation was performed by changing the thin films' orientations at 30°, 60° and 90° with respect to the incident beams. Remarkably, an enhancement of ∼6 times in RTFM, tuning in band gap from 3.27 to 3.21 eV and ∼60% reduction in surface roughness were noticed when the ion implantation was done at 60° to the normal. This novel technique may be suitable for tuning the physical properties of nanostructures for their application in the spintronics, semiconductor and solar cell industries.

## Abstract 2

The defects in the host lattice play a major role in tuning the surface roughness, optical band gap and the room temperature ferromagnetism of ZnO thin films.

## Graphical abstract

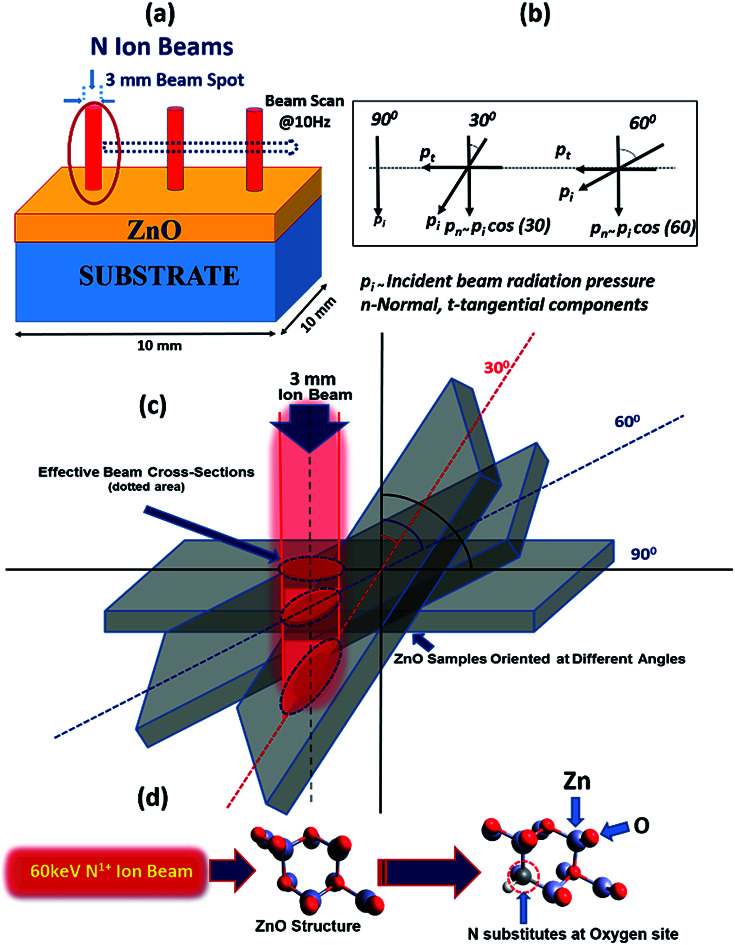

## 1. Introduction

The ion beam implantation technique has been widely used for the last two decades to tune material properties by generating defects in a very controlled and reproducible manner.^1–5^ In order to alter the physical properties, mainly the magnetic and optical properties of nanostructures by nonmagnetic ion implantation in metal oxides like ZnO, TiO_2_, SnO_2_, MgO, *etc.*, various research groups have extensively investigated varying the ion beam parameters such as current, energy, fluence and ion species.^6–10^ ZnO is selected due to its high exciton binding energy (60 meV), better resistance to radiation damage, high optical gain (320 cm^−1^) and wide band gap of ∼3.37 eV at room temperature.^11,12^ N ion beam implantation has an advantage over other dopants in producing shallow accepter levels with higher hole binding energy (∼400 meV) by replacing O ions in the ZnO nanostructure due to its ionic radius (∼1.46 Å) being comparable to oxygen (∼1.38 Å).^13–15^ In general, the room temperature ferromagnetism (RTFM) evolves in ZnO due to increase in the oxygen vacancies (V_o_) induced by the various defects like substitutions, interstitials, local structure transformations, *etc.*^16–20^ The defects induce the lattice distortion which results in the mechanical stress near dislocations and this leads to increase in band gap (compressional stress) or reduction (tensile stress) due to the forming of the bands and accumulating the defects. The incorporation of N induces the local lattice distortion due to the formation of pairs Zn_i_–N_o_ and Zn_i_–O_i_ which results in change in the polarity of Zn–O.^21–23^ This leads to the magnetism in nanostructures which needs to be controlled by tuning the defects in the host lattice. Pham *et al.*^24^ have reported the evolution of RTFM due to the substitution of N at O site in Zn–O nanostructure on the basis of *ab initio* study of spin-polarized total energy of various defects and nonmagnetic dopants having different charge states. They concluded that when N replaces O, it shortens the bond length of Zn–N due to the difference in N and O ionic radii. Jindal *et al.*^25^ have also investigated the RTFM by the substitution of N at the O sites in ZnO host lattice and concluded that the ferromagnetism may be controlled by varying the laser energy densities during thin film growth and it was mediated by the hole concentrations. In order to understand this phenomenon, more importantly the controlling of ferromagnetism in pure and N ion implanted ZnO nanostructures, many systematic efforts have been made till date but the origin of the ferromagnetism is still controversial and under discussion. Recently, it was reported that the tuning of RTFM and optical band gap have been carried out just by controlling the ion beam profiles and keeping other ion beam parameters constant.^26^ Further, the angle dependent implantation studies have been performed in nano-patterning with the realization of cascade collisions and mass redistributions on the surface produced by the transfer of energy and momentum of the incident charged particles under certain conditions of energy range, and fluence.^27^ In the present study, we report for the first time to control the ferromagnetism, band gap energy, surface roughness and grain size in N implanted ZnO nanostructured thin films by changing the implantation angles only and keeping other ion beam parameters such as current, energy, fluence and beam size constant.

## 2. Experimental details

The nanostructured ZnO thin films of ∼300 nm thickness were deposited on the silicon substrate by using the RF sputtering technique by applying the power of ∼200 W for half an hour at room temperature. The vacuum in sputtering chamber was maintained at ∼2 × 10^−2^ Torr during deposition under the pure Ar gas. No external heating was provided to the substrates. The ion implantations were performed by keeping 1 μA current of 60 keV N ion beam. This experiment was performed in material science beam line of Low Energy Ion Beam Facility at Inter University Accelerator Centre (IUAC), New Delhi. The ion beam spot size (3 mm), fluence (1 × 10^16^ ions per cm^2^) and energy (60 keV) were fixed. The structural modifications and crystal orientations were investigated by the X-ray diffraction (XRD) using Bruker D8 X-ray diffractometer with CuKα radiation (wavelength *λ* = 1.54 Å). The glancing angle was kept 3° in the detector scan mode. The machine was operated at 40 kV and 40 mA. The compositional analysis was carried out by using the Rutherford backscattering (RBS) spectrometry technique in the oxygen resonance (OR) mode (hereafter referred to as OR-RBS) using 1.7 MV Pelletron accelerator at IUAC with 3.05 MeV ^4^He^2+^ beam of 10 μC charge having energy resolution of 16 keV. The detector was kept at the angle of 166° with respect to the incident beam direction. The OR-RBS is suitable especially to give the information to estimate the oxygen content in the ZnO matrix. The ZnO nanostructures deposited on Si were used for OR-RBS studies, since the films on quartz or glass were not suitable for this study due to the presence of the inherent O in the substrates. The AFM (Nano-scope IIIa SPM II in tapping mode) was employed to study the morphological aspects whereas the optical properties (UV-visible) of implanted and non-implanted nanostructures were determined using HITACHI-3300 double beam spectrophotometer in the transmission mode. The VSM (VSM-5, TOEI Industry Co. Ltd. Tokyo, Japan) was used to study the ferromagnetic behaviour of the aforesaid nanostructures at room temperature with the magnetic field of ±2.0 T applied parallel to the surface of nanostructure. The sample was kept in the uniform magnetic field to produce the magnetization in the sample and then it vibrated sinusoidally in the presence of pick up coils. The variation in magnetic flux induces the proportional voltage signal in the pick-up coils. It measures the magnetic moment present in the sample with higher accuracy. The *I*–*V* characterization were performed to investigate the electrical properties and carrier concentration. Further, the magnetic switching behaviour and magnetic exchange coupling were investigated by Magnetic Force Microscopy (MFM).

## 3. Results and discussion

**Fig. 1:**
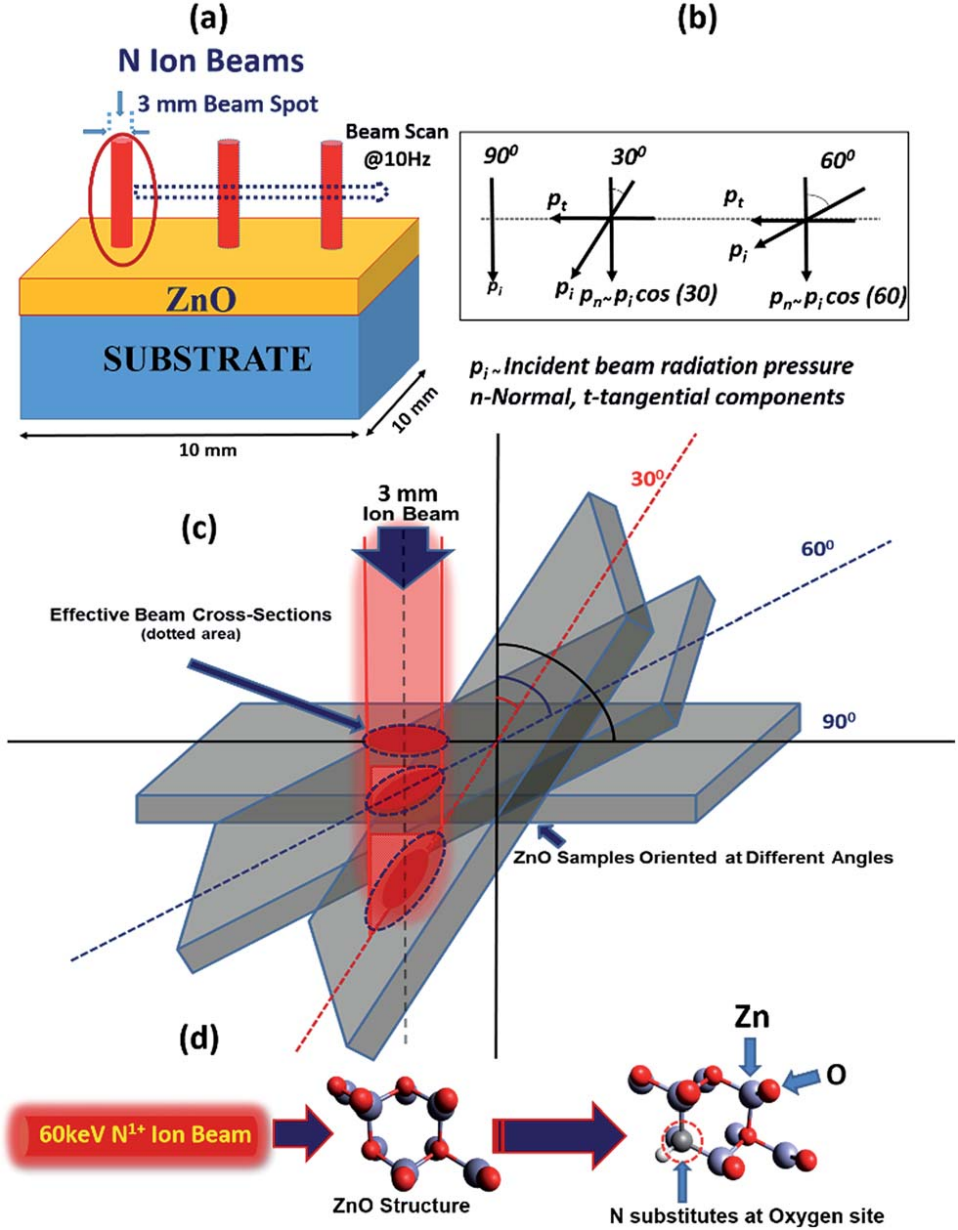
(a) Schematic of N beam scanning the area of 10 × 10 mm^2^ of samples at normal. (b) The incident beam radiation pressure and its associated normal and tangential components. (c) Effective beam cross section variation at different orientations of sample for 3 mm beam size. (d) Substitution of N at O site into ZnO lattice structure responsible for V_o_ defects.

In general, the normal incidence (90°) is used for the ion implantation studies. The pristine ZnO thin films and N ion beam implanted thin films at 30°, 60° and 90° hereafter will be referred to as ZnO and ZnO:N30, ZnO:N60 and ZnO:N90, respectively. These three angles were selected for the N ion implantations due to various considerations. A significant asymmetric variation is expected only at these three angles in view of the normal and tangential components of the radiation pressure exerted by the ion beam (these will be complementary to each other for the angles 30° and 60° and shall provide results worth for comparison). We have also selected the angle 90° where the normal component is maximum without any tangential component. For other angles (0° < angle of implantation < 90°), the normal and tangential components vary. In case of ZnO:N30, the normal component will be higher compared to tangential component. Similarly, the tangential component will be higher for ZnO:N60. For other angles, these components carry intermediate magnitudes. Hence, most of the information can be extracted based on these three angles. It is evident from the schematic in Fig. 1 that even though the beam energy (60 keV) and beam size (3 mm) are the same, the effective beam cross-sections acquired on the surface and projected range of ions in the lattice during the ion implantation at various angles are different. It is also taken care to make the fluence constant throughout the experiment by scanning the area of 10 × 10 mm^2^ by the N ion beam having constant current (1 μA) for constant time and scanning frequency (10 Hz).

**Fig. 2:**
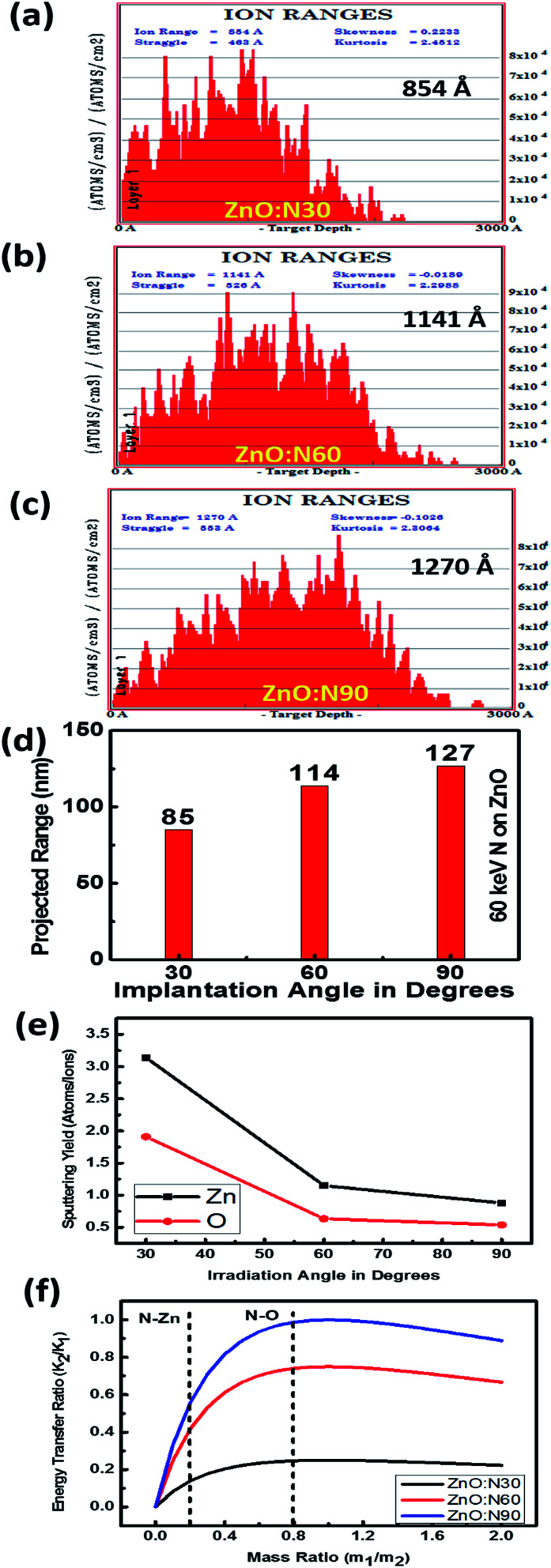
Atomic density distributions of (a) ZnO:N30. (b) ZnO:N60. (c) ZnO:N90. (d) Projected ion range *vs.* angle of ion implantations. (e) Sputtering yield *vs.* angle of ion implantation. (f) Energy transfer ratio for various ZnO:N samples.

In order to understand the angular sensitivity to the N ion beam implantation, the calculation of the atomic density, projection range, sputtering yield and energy transferred from the incident ion to the host lattice atoms were carried out by using SRIM software^28–30^ and are shown in Fig. 2.

It is evident that the energy transferred from N ion to ZnO is maximum in case of the normal incidence and when the beam has a longer projected range (∼127 nm) but it leads to the lowest sputtering yield. Further, the sputtering yield was found to be higher for the angle of 30° compared to the angle of 60°. Contrary to this, the energy transferred from N to O is ∼4 times higher at the angle of 60°. We have not chosen the implantation angle less than 30° because the ion implantations at those angles dominate more on the surface due to the higher sputtering yield (>5 atoms per ion) and lower energy transfer ratio from the incident ions to the surface atoms (<0.1). The ion interaction range was also found less than 80 nm which will reflect only the surface phenomena. Further, the thickness of the film is ∼300 nm and the energy of the incident ions (60 keV) is kept in such a way that the beam implantations take place efficiently up to the middle of the nanostructured thin films, *i.e.* ∼100–150 nm. In order to expedite the asymmetric distribution of the incident ions into the host lattice, we have chosen 30° and 60° ion implantations. The normal and tangential components of the energy transfer by the implanted ions are inhomogeneous in the case of these angles whereas it will be homogeneous in the case of angle 45°. Due to the similar effect at 45° from both the sides of the sample, this angle was also left.

**Fig. 3:**
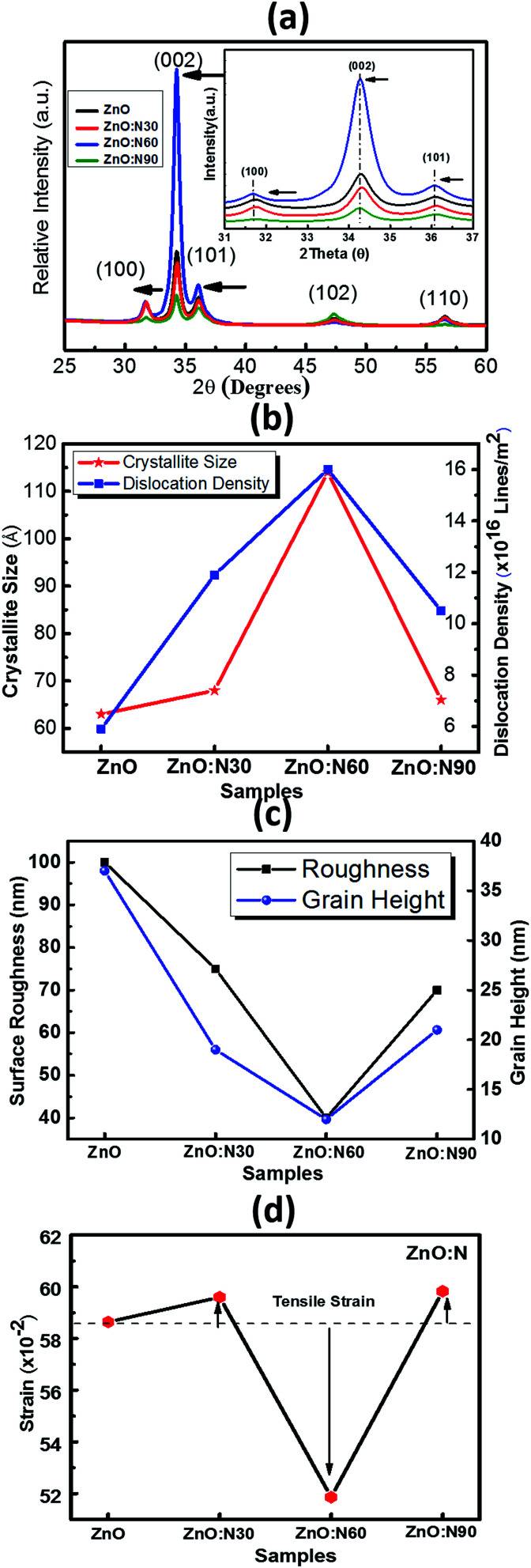
(a) XRD pattern of all the films. (b) Crystallite size and dislocation density. (c) Surface roughness and grain height. (d) Strain *vs.* various ZnO:N samples.

The XRD patterns of ZnO and N implanted ZnO nanostructures for the dynamic range of 2*θ* scanning from 25° to 60° degrees are shown in Fig. 3a. It is evident that these films were polycrystalline in nature and the coexistence of multi-peaks results in the grains formation in the crystal. Five major diffraction peaks were identified at 31.76°, 34.39°, 36.18°, 47.50°, 56.64° which correspond to (100), (002), (101), (102), and (110) planes, respectively. The high intensity peak along (002) plane corresponds to the crystal in hexagonal wurtzite (JCPDS card no. 79-0206) structure.^31^ When N replaces O site in the ZnO, it may result in the change in the stoichiometry of the nanostructure thin films and produces the tensile strain in the lattice due to the difference in ionic radii of N (2p_1/4_ 1.46 Å) and O (2p_1/4_ 1.38 Å) which leads to the expansion of crystallite size. Since ZnO nanostructured thin films are having large native point defects, it is expected that N ions can also go into the interstitial defect sites. However, there is a significant enhancement in the dislocation density, crystallite size and lattice parameters for ZnO:N60. A significant jump in the intensity of (002) peak and its narrowing improves the quality of crystals in polycrystalline ZnO nanostructures. The dominance of (002) peak over (100) and (101) for ZnO:N60 indicates that the preferential orientation of grain formations is in the (002) plane. The crystallinity improves with the formation of grains and grain boundary in the crystal after N ion implantations (Fig. 3b). The strain and the surface roughness were found to reduce for ZnO:N60 than the others, which results in the better crystallinity (Fig. 3c and d). A significant jump in the intensity of (002) peak and its narrowing shows the crystalline quality in the case of ZnO:N60. Its lowest surface free energy leads to the film growth along (002) plane. The high peak intensity and reduced FWHM of (002) peak represent the enhancement in the average crystallite size. The lattice planes (100), (002) and (101) were found to be shifted towards lower 2*θ* values due to the presence of tensile stress. This result in the significant variations in their lattice parameters and hence, the dislocation density.

**Fig. 4:**
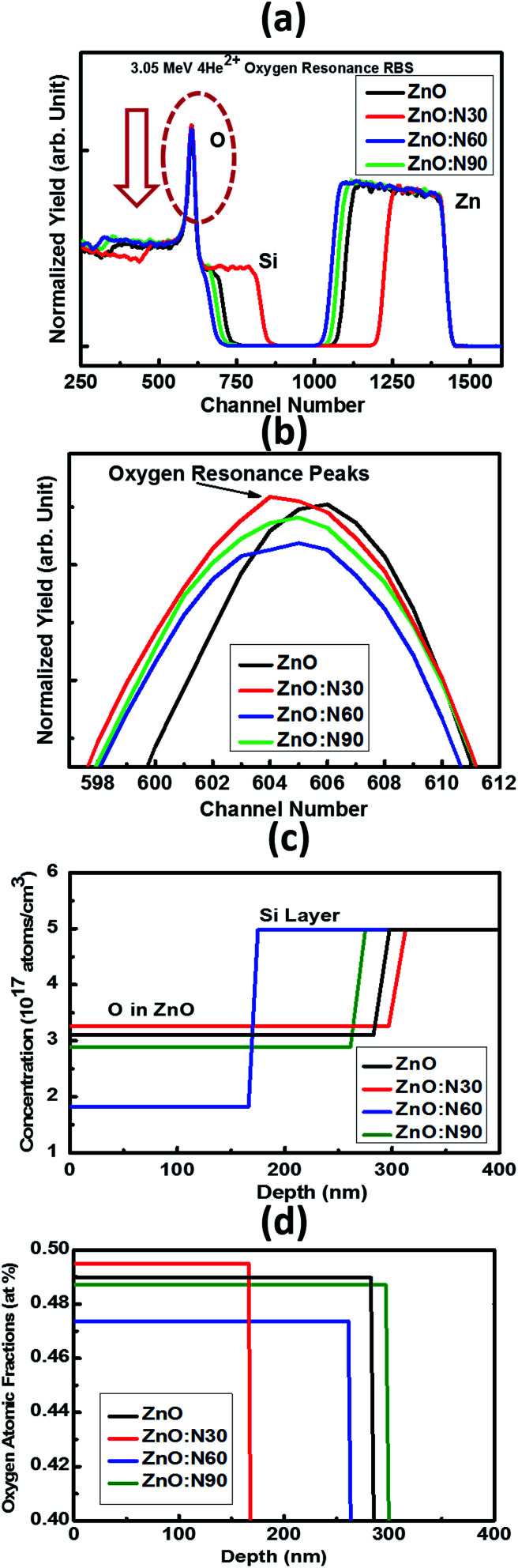
(a) OR-RBS spectra, (b) oxygen resonance peaks, (c) concentration and (d) oxygen atomic fractions *vs.* depth profiles for ZnO and ZnO:N samples.

It was very difficult to identify and estimate the variation of N content in the implanted ZnO matrix, especially at lower fluence of 1 × 10^16^ ions per cm^2^ using RBS due to the fact that these N and O ions are very light elements and these are neighbours in the periodic table. The RBS is relatively insensitive to light ions. So, it was decided to do the RBS in the oxygen resonance mode (OR-RBS) to confirm the N ions implantations in the ZnO films. Hence, the film thickness, stoichiometry and compositional analysis of N implanted ZnO were determined by OR-RBS technique. The data analysis and curve fittings were performed by data deconvolution program called Rutherford Universal Manipulation Program (RUMP).^32^ The OR-RBS and the RUMP simulated spectra for various samples are shown in Fig. 4. It is clearly seen in Fig. 4b that the oxygen peak intensity is comparable for ZnO:N30 and ZnO whereas it progressively reduced for ZnO:N90 and ZnO:N60 due to the substitution of N ions at the oxygen sites. The normalized yield was found lowest for ZnO:N60 due to the higher concentration of implanted N in the host lattice. It can be seen in Fig. 4c that O concentrations are the lowest of ∼1.8 × 10^17^ atom per cm^3^ for ZnO:N60 while it is the highest of ∼3.3 × 10^17^ atom per cm^3^ for ZnO:N30. The introduction of defects by substitution of N at the place of O during the implantation at 60° is more dominant due to the higher ion range and higher energy transfer ratio rather than at 30°. From Fig. 4d, it is evident that oxygen atomic fraction is the maximum for ZnO:N30 due to the higher sputtering yield and is the lowest for ZnO:N60. These results clearly show the variation in the concentration of O ions into the ZnO nanostructures due to the N ion implantations. This has validated the introduction of substitutional defects and formation of oxygen vacancies into the lattice system. The higher the defects, higher the magnetisation has been observed.

**Fig. 5:**
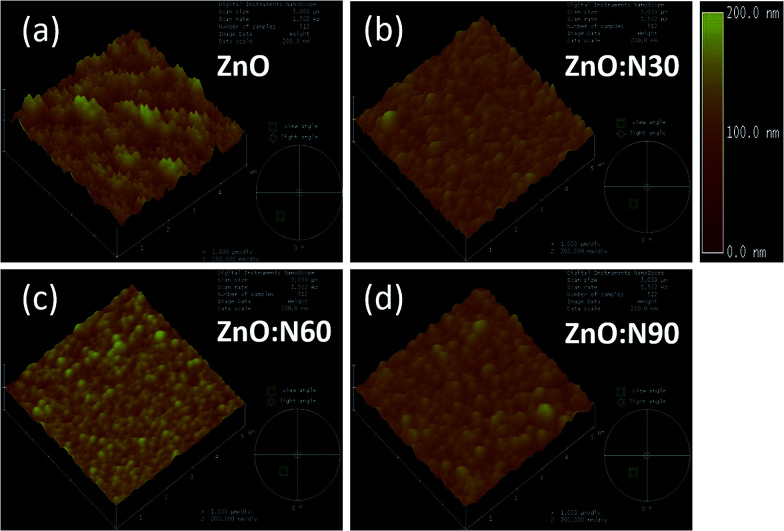
AFM images of (a) ZnO. (b) ZnO:N30. (c) ZnO:N60. (d) ZnO:N90 samples.

To understand the morphology of the N implanted nanostructures at various angles, the AFM measurement was performed and the observed images are depicted in Fig. 5. In general, the surface roughness depends mainly on the incident angle if the other ion beam parameters are kept the same. Although the ion energy and fluence are kept constant, the sputtering yield and incident ion range in the host lattice may vary with the incidence angles. As is evident from the AFM images, there is a maximum reduction (∼60%) in the roughness and ∼70% reduction in the grain height (Fig. 3c) for ZnO:N60 than the ZnO. The ion range in the host lattice and the sputtering yield for Zn (O) for 60 keV N ion implanted at 60° were evaluated to be ∼110 nm and ∼1.2 (∼0.6) atoms per ion, respectively, by using SRIM/TRIM softwares.^28^ The sputtering yield at 60° is moderate and removes the chunk of atoms from the hills and fills the valley on the surface, resulting in smoother surface than ZnO:N30 and ZnO:N90. The sputtering yield at 30° is the highest for Zn (∼3.4 atoms per ion) and O (∼1.9 atoms per ion) which results in ∼35% less smoothing effect on the surface than ZnO:N60 samples. It can be well understood that the surface smoothing effect of the nanostructure thin films strongly depends on the angle of ion implantation.

**Fig. 6:**
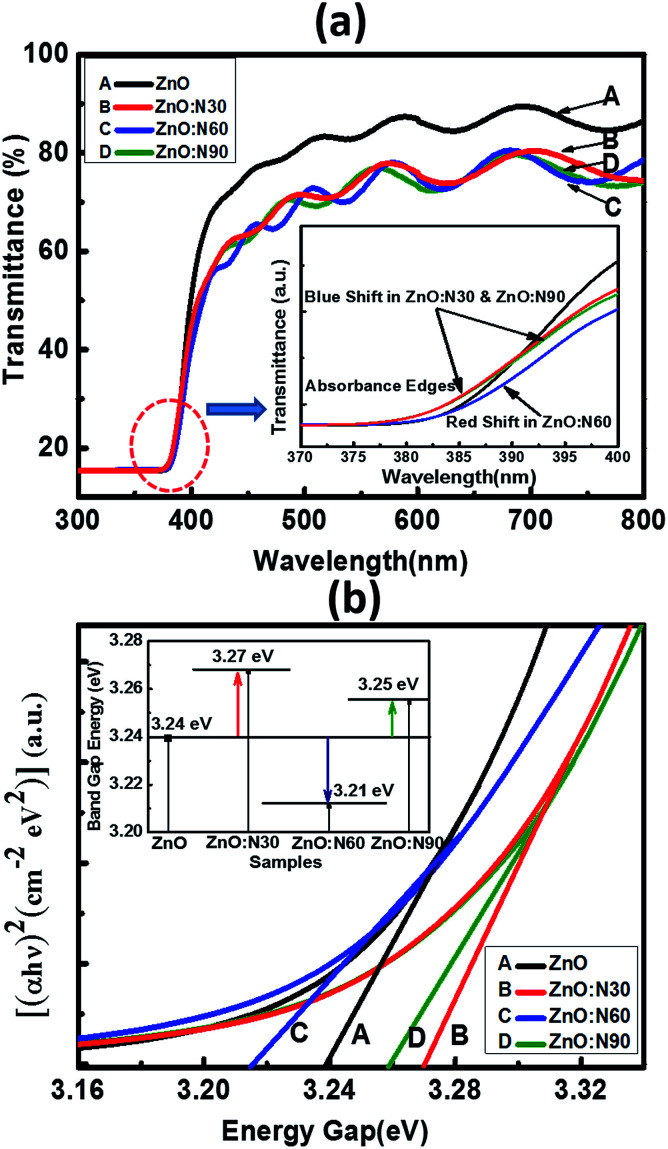
(a) Transmittance with enlarged region of the absorption edges. (b) Tauc's plot equipped with band gap tuning.

In order to further study the optical properties of aforementioned samples, UV-vis characterizations were performed for the wavelengths ranging from 200 to 800 nm and the results are shown in Fig. 6. The optical transmission spectra of ZnO and ZnO:N implanted at various angles is shown in Fig. 6a. It is evident that the ZnO:N nanostructure thin films are transparent in nature in the visible region and exhibit a fundamental absorption edge at ∼380 nm. It was observed that ZnO yields to the highest transmittance (∼87%) in the visible region whereas the reduction in its transparency has been found to be ∼20% for ZnO:N sample. The lowest transmittance was obtained to be ∼72% for ZnO:N60. In order to correlate the band gap variation with the absorbance edges in the transmittance spectra, we have found that the absorption edge of ZnO:N60 has a clear shift towards the higher wavelength (red-shift) that results in the band gap reduction. The absorption edges for ZnO:N30 and ZnO:N90 shift towards the lower wavelengths (blue-shift) and hence, increases the band gap of the nanostructures.

The N ion implantation in ZnO lattice modifies the band gap and also the shallow accepter/donor level states in the forbidden gap by the incorporation of N on O site. The mixing of shallow N 2p states with the valence band and conduction band of ZnO lead to the reduction and enhancement of band gaps, respectively.^33–35^ The band gap was calculated by employing the linear extrapolations of Tauc's plot.^36^ The ZnO:N30 contains higher order of non-stoichiometry in the lattice which changes the lattice structure in such a way that their band edges move farther due to the higher rate of bending of their near band edges and the Fermi level (*E*_f_) shifts towards the conduction band, which leads to the enhancement of band gap (3.27 eV) (Fig. 6b). Furthermore, it was observed from the SRIM/TRIM calculations that the sputtering yield is higher for ZnO:N30 than the others. The higher order of sputtering yields result in the accumulation of defect on the surface and hence, the higher concentration of surface defects. This higher density of defects is attributed to the higher number of O adsorbed on the surface and results in the larger band gap. Contrary to this, ZnO:N60 leads to decrease in the band gap energy. The higher order of mechanical stress near dislocations due to implantation at 60° could be the reason for the band gap reduction due to the formation of N_2_O bands which acts as a source of O as well as N. This may increase the 2p-states in the valence band due to an increase in the Zn–N bonds and a decrease in V_o_ in the host lattice, by which their band edges come closer to each other and *E*_f_ shifts towards the valence band and leads to the reduction in band gap (3.21 eV). So, mainly the overlapping of N 2p and O 2p states may be the cause of significant band gap reduction in ZnO:N60.^37,38^

**Fig. 7:**
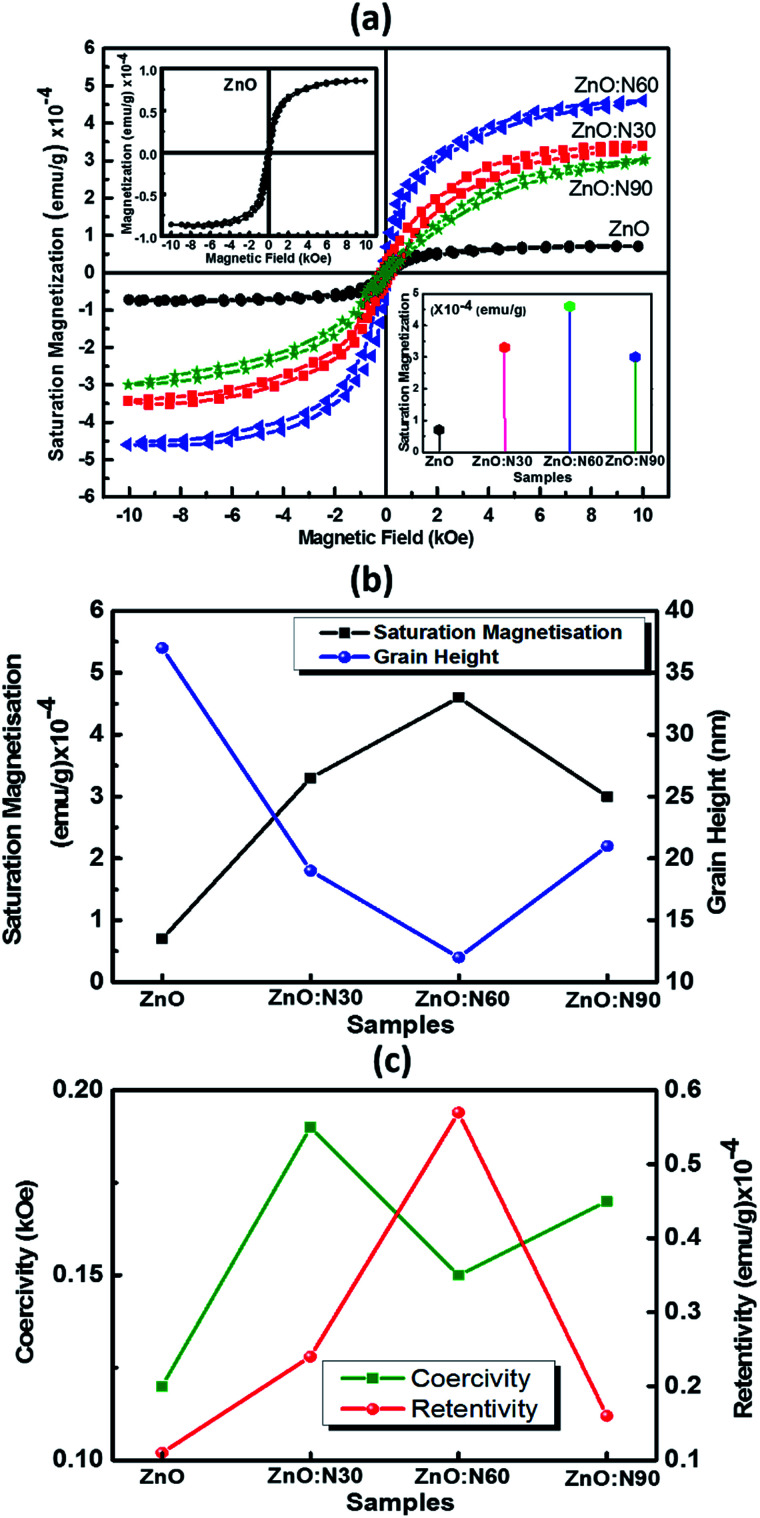
(a) Saturation magnetization curves *vs.* applied external magnetic field, (inset-I for ZnO and inset-II for magnetization magnitude at different angles). (b) Saturation magnetisation *vs.* grain height. (C) Coercivity and retentivity for various samples.

Recently, nanostructured materials have been investigated extensively to enhance the ferromagnetic capability in nonmagnetic materials. Fig. 7 shows the saturation magnetizations for pristine and N implanted ZnO at various angles. The saturation magnetisation curves have been plotted after subtracting the diamagnetic part from the substrate Si signal. The signature of the grains formation and grain boundaries into the lattice are also the reasons for the evolution of the RTFM. The N ion implantations at 60° orientations result in the reduced strain and enhanced dislocations density in the nano-regime of ZnO lattice. This is the most favourable state to strengthen the magnetic coupling at this angle of implantations. This leads to higher magnetic moments into the host lattice and hence, results in the maximum saturation magnetization. In addition, smallest sized grains were also obtained in the case of ZnO:N60 films. It is evident that a very weak saturation magnetization (0.7 × 10^−4^ emu g^−1^) is present in ZnO due to native defects, impurities and hydrogen bonding introduced during the thin film deposition. The order of magnetization depends on the deposition conditions during RF sputtering in the vacuum chamber. A well-defined magnetic hysteresis loops have also been obtained for ZnO:N30, ZnO:N60 and ZnO:N90 which confirm the ferromagnetism at room temperature (Table 1).

**Table 1:** Physical parameters of ZnO and ZnO:N for three different implantation angles

Physical properties	ZnO	ZnO:N30	ZnO:N60	ZnO:N90
Magnetization (emu g^−1^) × 10^−4^	0.7	3.3	4.5	3.0
Coercivity (kOe)	0.12	0.19	0.15	0.17
Retentivity (emu g^−1^) × 10^−4^	0.11	0.24	0.57	0.16
Magnetic phase (°)	0.9	2.8	3.9	2.6
Transmittance (±1%)	86	77	71	73
Band gap (±0.05 eV)	3.24	3.27	3.21	3.25
Surface roughness (nm)	98	74	38	67
Grain height (nm)	37	19	12	21
Dislocation density (10^16^ lines per m^2^)	6	12	16	10

The saturation magnetization of ZnO:N is found to enhance by 4–6 times by varying the implantation angles by keeping the ion fluence, current, energy and incident beam size constant. These results deduce that the extreme sensitivity of the ferromagnetism is not only to the nature of N induced defects in the ZnO nanostructure but also the implantation angle. The saturation magnetization for ZnO:N60 is the maximum of ∼4.5 × 10^−4^ emu g^−1^, for ZnO:N30 is ∼3.3 × 10^−4^ emu g^−1^ and for ZnO:N90 it is 3 × 10^−4^ emu g^−1^. There is a significant change in the spin polarization and magnetic moment with N ion implantation in ZnO and the saturation magnetization also varies four folds in ZnO:N with angle of implantations. Defects and vacancies near the grain boundaries are created by N ion implantation and this varies with angles in the present study and enhances the magnetic exchange interaction between π- and σ-bonds in the three-dimensional structure.^39^

At a first glance, when N is incorporated in ZnO, there is a formation of relatively shallow accepter, and its efficiency depends on the local environment and impurities during the implantation.^40,41^ As is evident from the XRD patterns, the ZnO:N60 has higher dislocation density and higher order of strains in the host lattice that results in the higher density of singly charged O vacancy defects and hence, introduces the stronger polarization field.^42,43^ This higher density of V_o_ defects states produces the higher order of unpaired states and most probably results in the higher order of ferromagnetism at room temperature in ZnO:N60 nanostructured thin films. Further, the ferromagnetism diminishes in ZnO:N30 not only due to the low strain but also increase in the local temperature during implantation. The effective beam cross section is higher in ZnO:N30. Due to overlapping of beams, there is an increase in surface energy in ZnO:N30 which is higher compared to the others (ZnO:N60 and ZnO:N90). This results in the low V_o_ defect states than the ZnO:N60. Variation of implantation angle significantly changes the effective cross section of the beam and this may also lead to change in thermal instability and saturation magnetization of the host lattice due to higher order anisotropic distribution of N ions.^44^ The signature of grains formation and grain boundaries into ZnO:N nanostructure also cause the evolution of this magnetism.^45–47^

It was also observed from the SRIM calculations that the ion ranges clearly depend on the angle of implantations. It is also found that the sputtering yields for Zn and O ions are higher for ZnO:N30 (Zn ∼3.4 atoms per ion and O ∼1.9 atoms per ion) compared to the other samples (ZnO:N60 and ZnO:N90). Higher order of sputtering yields results in the accumulation of defects on the surface and hence, the higher concentration of surface defects are present in the ZnO:N samples. The RTFM is observed in the N implanted ZnO thin films due to the formation of vacancies, grain boundaries and crystallographic imperfections in the crystal structure and these vary with the angle of implantations. The grain boundaries and oxygen vacancies are the intrinsic origin for RTFM. Theoretical investigations have clarified the importance of O vacancies and grain boundaries for long range magnetic interactions and ferromagnetism. Some studies have shown that grain size variation and oxygen vacancies at the surface of nanostructures are dominant factors for the generation of such magnetism. The bombardment of N ions on ZnO surface induces the magnetic moments between N and O ions due to the p–p interaction coupled to the host matrix and the magnetization varies with the angle of implantation.^48,49^ In general, more defects refer to more magnetisation as well as to higher surface roughness. But it was observed that the N implanted ZnO thin films in our case results in the smooth surface with higher magnetisation and this makes our samples more applicable and advantageous than those of the others. Straumal *et al.* showed that the magnetisation was observed in the presence of a magnetic atoms (Mn) whereas we have implanted a non-magnetic element N into the ZnO host lattice to produce the RTFM.^20^

**Fig. 8:**
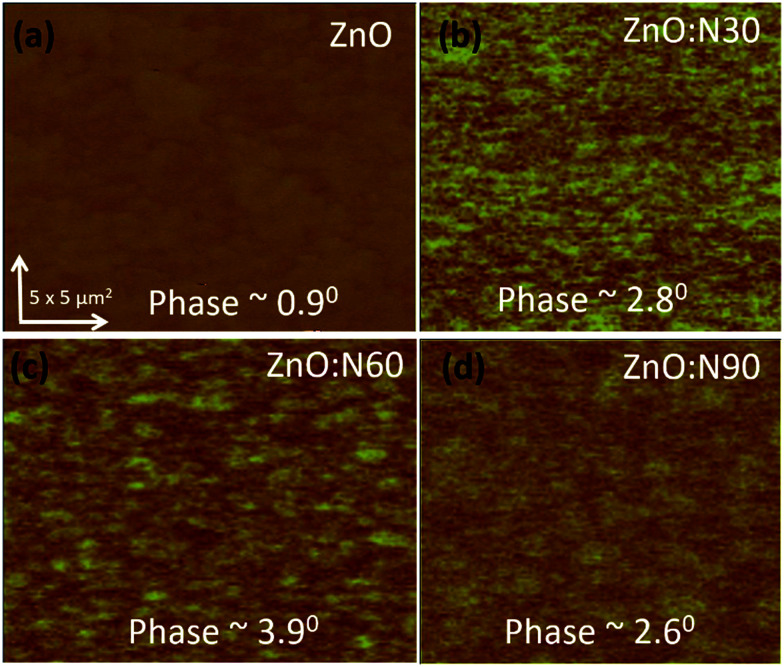
MFM images of (a) ZnO. (b) ZnO:N30. (c) ZnO:N60. (d) ZnO:N90 samples.

In order to understand the mechanism of ferromagnetism at room temperature, MFM analysis was also performed. Both AFM and MFM were carried out at the same position and the height. Fig. 8 shows different MFM images obtained at various implanted angles. The N ion implantation at different angles results in the formation of magnetic domain cluster and the size of cluster depends on the magnetic exchange coupling among the grains in the nanostructure. The domains obtained are formed by magnetic interactions of the neighbouring grains. The higher order of magnetic exchange coupling is observed in ZnO:N60 due to the formation of larger domain cluster and hence, there is a maximum change in the magnetic phase (∼3.9°). The phase magnitude reduces for ZnO:N30 and ZnO:N90 due to the smaller magnetic domain. This results in less exchange coupling among the grains.

**Fig. 9:**
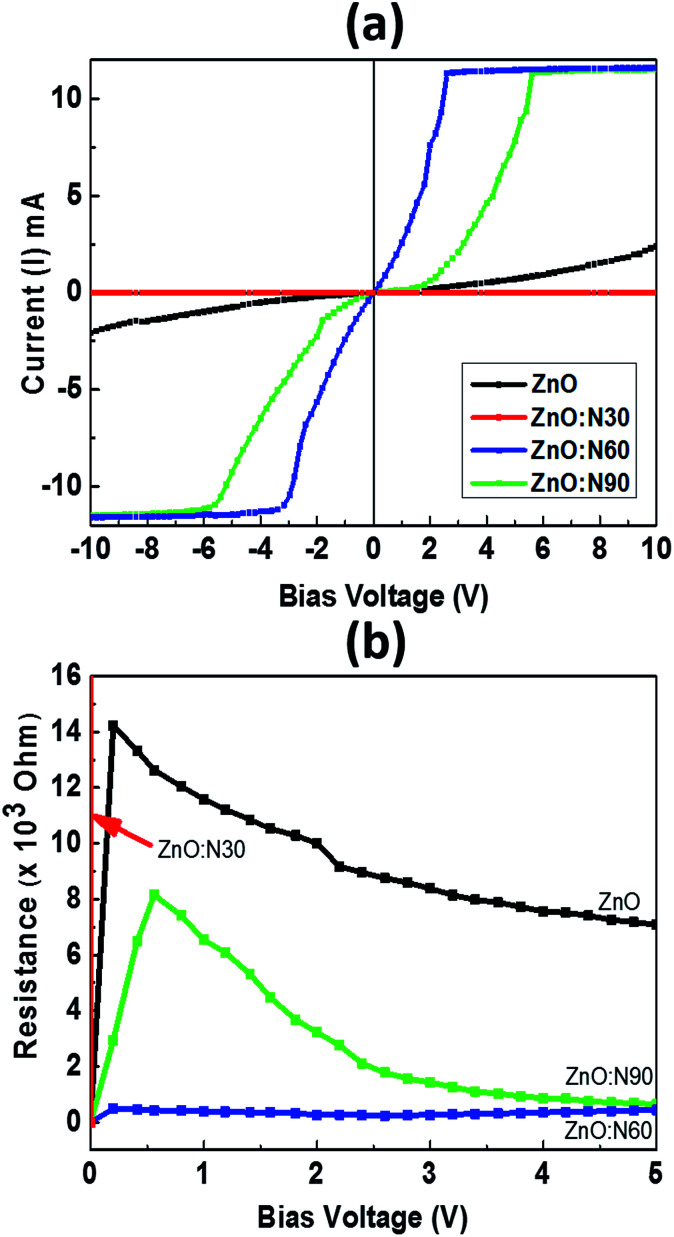
(a) *I*–*V* graph. (b) Resistance *vs.* bias voltage for pure and N implanted samples.

Fig. 9 depicts the *I*–*V* characteristics of ZnO, ZnO:N30, ZnO:N60 and ZnO:N60. It was observed that the *I*–*V* curves vary non-monotonically over a large dynamic range and exhibit non-linear and symmetric behavior for a certain range of bias voltage from −10 to +10 V. These show a very interesting transition from larger span of bias voltage in ZnO:N90 to lower as in ZnO:N60. The resistance was also calculated for ZnO and ZnO:N. In Fig. 9b, the dominant *I*–*V* characteristics for ZnO:N60 gives rise to a low resistance of approximately 1 kΩ. The conductivity changes due to the enhancement of hole concentration induced by the incorporation of N into ZnO lattice. The implantation of N ions alters the local electronic structure in the nanostructure due to the overlapping of π* and π bands near Fermi energy level. The terminating edge on the surface introduces the sharp discontinuities in *I*–*V* characteristics. The current increases linearly with increasing applied bias voltage within a certain range, however, this dependence does not appear when the applied bias voltage exceeds a certain value. The current increases linearly when the bias voltage increases from −3 V to 3 V before saturating at voltages above ±3 V as in the case of ZnO:N60 and this linear behavior is further strengthened for other samples. In the linear current regime, the conductance is found to be higher for ZnO:N60 based on the slopes of the *I*–*V* graph. Based on the previous results it is found that when N substitutes O in ZnO, N–O pair could be responsible for shallow accepters that couples with the hydrogen atoms. Moreover, the interaction between H (donor) and N–O (accepter) could push the levels closer to the valence band from deep accepter levels. Further, pairs of N_O_–V_Zn_ have also shown as a shallow accepter which results in the higher conductance in the matrix. The implantation of N into ZnO lattice at low energy (60 keV) led to the variation in band gap energy also for ZnO:N samples due to the presence of shallow accepter/donor level states in the forbidden gap by the incorporation of N on O site. The higher order of mechanical stress is seen in ZnO:N60 which may be responsible in the reduction of the band gap and hence, the enhancement of conductance due to the formation of N_2_O bands which act as a source of O as well as N.^49^ This can be ascribed mainly by overlapping of N 2p and O 2p states which may be responsible for the enhancement of conductance in ZnO:N60. The electrical conductivity of N implanted ZnO can also be understood by the quantum surface effect. The scattering of the internal as well as the external electrons of the surface atoms are responsible for the electrical resistance for ZnO:N where the density of states are varying due to the quantization of Fermi level.

## 4. Conclusions

Present study reveals the significance of implantation angle to control the ferromagnetism, band gap and surface roughness in nonmagnetic ion (N) doped ZnO nanostructured thin film in a very precise manner. Herein, incorporation of N at 60° in ZnO induces the stronger ferromagnetism, higher band gap reduction, larger reduction in surface roughness and higher reduction in the grains at room temperature. A smaller band gap can absorb the longer wavelength (visible) light whereas the smaller grains could enhance the magnetic storage capability. Thus, the smoother surface achieved with smaller band gap not only absorbs longer wavelength but also significantly enhances its light absorption capacity in the visible region with less reflection.

## Conflicts of interest

There are no conflicts to declare.

## Supplementary Material
